# The Shape of Care: Patterns of Family Caregiving Among Chinese Adults in the Middle to Later Stage of Life

**DOI:** 10.1093/geroni/igab046.2930

**Published:** 2021-12-17

**Authors:** Haoshu Duan

**Affiliations:** University of Maryland, Arlington, Virginia, United States

## Abstract

Due to the lack of institutional support, families have long been the primary caregivers in China. Most studies to date only focused on one single care activity during a particular life course stage. Nonetheless, older adults today are more likely to care for multiple family members concurrently or sequentially (serial caregivers). The studies on discrete snapshots of care activities failed to capture the patterns of family caregiving overtime. Utilizing four waves of longitudinal data from CHARLS (2011-2018, N=17,039), this study particularly focuses on care activities to grandchildren, parents, and spouse, and maps out the family caregiving patterns overtime. Using latent profile analysis, this study identifies five family caregiving patterns: 1). Light grandchild caregivers (27%), who on average provided 4.3 years’ grandchild care mostly; 2). Heavy grandchild caregivers (11%), who on average on provided 7 years’ grandchild care mostly; 3). Light caregivers for grandchildren and parents (7%), who sequentially provided 1-year care to grandchildren and parents; 4). Heavy serial caregiver (6%), who mostly provided care to spouse and grandchildren with higher overlapping years; 5). Overall light caregivers (49%), who on average provided less than one year of care to any recipient. The preliminary results suggest that heavy serial caregivers (6%) far worst in terms of depressive symptoms and more likely to report worsened self-rated health; and overall light caregivers (49%) have the lowest depressive symptoms and more likely to report good self-rated health.

